# Progranulin and Its Receptor Predict Kidney Function Decline in Patients With Type 2 Diabetes

**DOI:** 10.3389/fendo.2022.849457

**Published:** 2022-04-01

**Authors:** Maki Murakoshi, Tomohito Gohda, Hiroko Sakuma, Terumi Shibata, Eri Adachi, Chiaki Kishida, Saki Ichikawa, Takeo Koshida, Nozomu Kamei, Yusuke Suzuki

**Affiliations:** ^1^ Department of Nephrology, Faculty of Medicine, Juntendo University, Tokyo, Japan; ^2^ Department of Endocrinology and Metabolism, Hiroshima Red Cross Hospital and Atomic-Bomb Survivors Hospital, Hiroshima, Japan

**Keywords:** progranulin (GRN), tumor necrosis factor receptor (TNFR), biomarker, renal outcome, diabetic kidney disease

## Abstract

Progranulin (PGRN), a growth factor, is abundantly expressed in a broad range of tissues and cell types with pleiotropic functions including inflammation, neurodegeneration, and facilitating lysosome acidification. PGRN binds to TNF receptors (TNFR) and inhibits downstream inflammatory signaling pathways. TNFR is a well-known predictor of glomerular filtration rate (GFR) decline in a variety of diseases. Therefore, we measured circulating PGRN in addition to TNFR using an enzyme-linked immunosorbent assay and explored whether it predicted renal prognosis in 201 Japanese patients with type 2 diabetes. During a median follow-up of 7.6 years, 21 participants reached primary renal endpoint, which involves a decline of at least 57% in eGFR from baseline, or the onset of end-stage renal disease. Univariate Cox regression analysis revealed that classical renal measures (GFR and albuminuria), two TNF-related biomarkers (PGRN and TNFR), and BMI were associated with this outcome. Multivariate analysis demonstrated that high levels of PGRN [HR 2.50 (95%CI 2.47–2.52)] or TNFR1 [HR 5.38 (95%CI 5.26–5.50)] were associated with this outcome after adjusting for relevant covariates. The high levels of PGRN as well as TNFR1 were associated with a risk of primary renal outcome in patients with type 2 diabetes after adjusting for established risk factors.

## Introduction

Diabetic kidney disease (DKD) is the most common cause of end-stage renal disease (ESRD) in Japan (1, 2). It is diagnosed by the presence of persistently increased albuminuria and/or a decreased estimated glomerular filtration rate (eGFR). Currently, these two measures are used as prognostic biomarkers of kidney function decline in chronic kidney disease (CKD) patients. However, previous studies indicated that albuminuria staging is inaccurate for the prediction of kidney function (3, 4). In fact, some studies have reported that DKD may progress without significant albuminuria (5, 6). Therefore, studies on alternative DKD markers other than albuminuria and eGFR are greatly needed (7).

Progranulin (PGRN), also known as granulin-epithelin precursor (GEP), proepithelin, acrogranin, and GP88, is a growth factor composed of 593 amino acids and is abundantly expressed in a broad range of tissues and cell types. As a unique pleiotropic factor, PGRN is involved in many cellular processes and diseases such as embryogenesis, tumorigenesis, inflammation, wound repair, neurodegeneration, and lysosome function (8).

PGRN plays an anti-inflammatory role in various inflammatory diseases, including rheumatoid arthritis (RA) (9), Alzheimer’s disease (10), atherosclerosis (11), and bacterial pneumonia (12), *via* its competitive binding to tumor necrosis factor (TNF) receptors (TNFR1 and TNFR2) (13) and interferes with the interaction between TNFα and TNFR.

We found that circulating levels of TNFR were robust predictors of GFR decline in patients with various stages of both type 1 and 2 diabetes (14, 15). Since then, prominent clinical application of the circulating TNFR level as a predictor of future GFR decline has been reported in a variety of diseases so far (16–18). Several studies have shown that serum levels of PGRN, a ligand of TNFR, were elevated in patients with diabetes and a decline in renal function (19, 20). Our cross-sectional study also reported a negative correlation between serum PGRN and eGFR in patients with type 2 diabetes (21). However, cross-sectional studies cannot be used to infer causality. Therefore, this study aims to investigate whether PGRN levels can also be used to predict renal prognosis in patients with type 2 diabetes.

## Material and Methods

### Patients

Between November 2011 and December 2020, we enrolled 264 patients with type 2 diabetes mellitus who were ≥ 20 years of age at Juntendo University Hospital in Tokyo, Japan. They were required to have eGFR, as calculated by the equation defined by the Japanese Society of Nephrology, of ≥30 ml/minute/1.73 m^2^ of body-surface area, more than 2 years of follow-up, and ability and willingness of the subject to cooperate with the study protocol. The final sample size included 201 participants. This study was approved by the Institutional Review Board of Juntendo University, Tokyo, Japan (document no. 778). All participants provided written informed consent, and this study complies with the ethical principles of the Declaration of Helsinki.

### Sample Collection and Laboratory Measurement

Blood samples were obtained at the time of registration and rapidly centrifuged at 4°C. Supernatants were transferred to RNase-free tubes and stored at a temperature of −80°C until utilized in subsequent assays. We used an enzyme-linked immunosorbent assay (ELISA) to measure PGRN and TNFR1 (cat. No. DPGRN0, DRT100; R&D Systems, Minneapolis, MN, USA) following a previously described method (21–23).

### Outcomes

The primary renal composite outcome of this study was the first occurrence of any of the following: a decline of at least 57% in eGFR from baseline (i.e., the serum creatinine level doubled from the baseline), or the onset of ESRD (maintenance dialysis, renal transplantation, or an eGFR <15 ml/minute/1.73 m^2^). The secondary outcome was all-cause death.

### Statistical Analyses

All variables were expressed as percentages for categorical data, and as the means ± standard deviation (SD) or median and interquartile ranges for continuous data with or without a normal distribution, respectively. Patients were stratified according to their quartile PGRN and TNFR1 levels for analytical purposes. The event cumulative risk was derived from the Kaplan–Meier method. The Cox proportional hazards model was used to identify independent predictors for developing a primary or secondary endpoint. The hazard ratio (HR) for the risk of a composite with 1 SD increase of logarithmic transformation of each protein was computed by Cox regression after adjusting for age, sex, body mass index (BMI), baseline eGFR, and the urine albumin-to-creatinine ratio (UACR). Pearson’s correlation coefficient was employed to test the correlations between different variables. Before testing the correlations, all non-normally distributed values were log-transformed to better approximate normal distributions. Next, stepwise multiple linear regression was performed to determine the factor that affects the serum PGRN level. A *P* value of less than 0.05 was considered to indicate a statistically significant difference. Analyses were performed using SAS software, version 9.4 (SAS Institute, Cary, NC, USA).

## Results

### Baseline Characteristics

The baseline characteristics of the study patients are presented in **Table 1**. The mean age was 67 years, and 73.6% of the patients were male. The mean glycated hemoglobin value was 7.2%; the mean BMI was 24.1 kg/m^2^; the median eGFR was 73 ml per minute per 1.73 m^2^; and the median UACR was 23, with albumin measured in milligrams and creatinine in grams. The median follow-up was 7.6 years (minimal 2.1, maximal 9.1 years, interquartile range 4.9–8.4 years). A total of 120 (59.7%) and 67 (33.3%) patients were treated with renin-angiotensin system (RAS) blockers and insulin therapy, respectively.

**Table 1:** Clinical characteristics and circulating PGRN concentrations in the study patients.

	Total (n = 201)	PGRN		p
		Below median (n = 100)	Above median (n = 101)	
Male (%)	73.6%	79.0%	68.3%	0.09
Age (yr)	67 ± 10	68 ± 11	67 ± 10	0.77
BMI (kg/m^2^)	24.1 ± 3.5	23.8 ± 3.5	24.5 ± 3.4	0.11
SBP (mmHg)	129 ± 16	131 ± 17	128 ± 14	0.25
DBP (mmHg)	71 ± 11	72 ± 11	71 ± 10	0.93
RAS blockers Tx (%)	59.7%	52.0%	67.3%	0.03
Smoker (%)	32.3%	38.0%	26.7%	0.12
Diabetes duration (yr)	17 ± 9	19 ± 9	16 ± 9	0.048
HbA1c (%)	7.2 ± 1.0	7.1 ± 0.9	7.2 ± 1.0	0.79
Insulin Tx (%)	33.3%	31.0%	35.6%	0.46
Uric acid (mg/dL)	5.4 ± 1.4	5.3 ± 1.4	5.5 ± 1.5	0.26
eGFR (mL/min/1.73m^2^)	73 (55, 91)	77 (64, 92)	62 (45, 87)	<0.01
UACR (mg/gCr)	23 (8, 142)	13 (6, 61)	44 (12, 421)	<0.001
CRP (mg/dL)	0.205 ± 0.620	0.080 ± 0.128	0.331 ± 0.853	<0.01
TNFR1 (pg/mL)	1,412 (1,153, 1902)	1,273 (1,093, 1,536)	1,629 (1,230, 2,301)	<0.0001
PGRN (ng/mL)	59 (52, 68)	52 (45, 56)	68 (64, 77)	<0.0001

Data are presented as the mean ± SD, median (quartiles), or %.

BMI, body mass index; SBP, systolic blood pressure; DBP, diastolic blood pressure; RAS, renin-angiotensin system; Tx, therapy; GFR, glomerular filtration ratio; UACR, the ratio of albuminuria to creatinine; CRP, C-reactive protein; TNFR, TNF receptor; PGRN, progranulin.

The median serum PGRN was 59 (52–68) ng/mL in all samples. Stratifying by the PGRN median in two groups, patients with above the median of PGRN were more likely to be associated with low eGFR, high UACR, and high C-reactive protein (CRP).

### Variables Associated With Circulating PGRN or TNFR1

The associations of clinical parameters with serum PGRN or TNFR1 levels are presented in **Tables 2** and **3**. According to Pearson’s correlation analysis, the serum level of PGRN was correlated with BMI, CRP, eGFR, and UACR. The association of eGFR, UACR, and BMI with PGRN was maintained in the stepwise multiple regression analysis. On the other hand, the serum level of TNFR1 was correlated with male, age, uric acid, CRP, eGFR, and UACR according to Pearson’s correlation analysis, and the association of eGFR, UACR, and CRP with TNFR1 was maintained by multiple regression analysis. A mild correlation was observed between the serum levels of PGRN and TNFR1 (r = 0.38, p <0.0001).

**Table 2:** Pearson’s correlation coefficients between serum PGRN or TNFR1 concentration and various clinical parameters in patients with type 2 diabetes.

	PGRN		TNFR1	
	r	p	r	p
Male	0.13	0.06	−0.13	0.04
Age	0.001	0.99	0.02	0.04
BMI	0.18	0.01	0.06	0.43
SBP	−0.02	0.74	0.04	0.56
DBP	0.01	0.90	−0.13	0.07
HbA1c	0.10	0.15	−0.04	0.62
Uric acid	0.14	0.06	0.47	<0.0001
CRP	0.16	0.03	0.18	0.01
eGFR	−0.28	<0.0001	−0.79	<0.0001
UACR	0.33	<0.0001	0.67	<0.0001
TNFR1	0.38	<0.0001	–	–

Abbreviations used in this table are the same as in **Table 1**.

**Table 3:** Stepwise multiple regression analysis of factors associated with serum PGRN or TNFR1 concentration.

Variable^#^	PGRN		TNFR1	
	*β*	*F*	*β*	*F*
eGFR	−0.17	4.72*	−0.60	281.10***
UACR	0.24	21.14**	0.40	94.08***
BMI	0.16	4.79*	–	–
CRP	–	–	0.08	4.71*
*R^2^*	0.15		0.75	

^#^Variables include eGFR, UACR, BMI, and CRP for PGRN. Variables include age, sex, uric acid, eGFR, UACR, and CRP for TNFR1. F value > 4.0 was considered significant.

*p < 0.05. **p < 0.01. ***p < 0.001.

Abbreviations used in this table are the same as in **Table 1**.

### Association of Clinical Factors and Biomarkers With Outcome: Univariate Analysis

**Figure 1:**
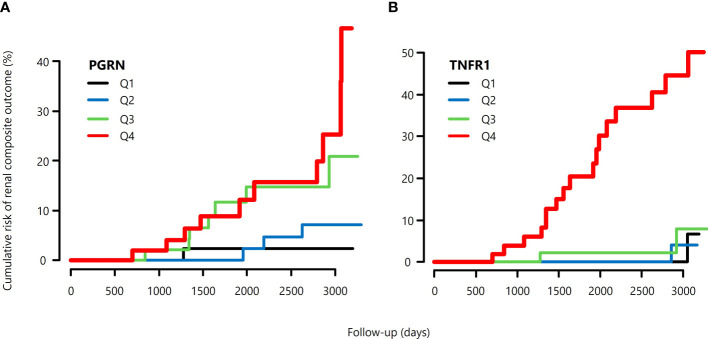
Cumulative risk of primary composite renal outcomes of patients with type 2 diabetes, **(A)** according to the quartile of serum PGRN at baseline, and **(B)** according to the quartile of serum TNFR1 at baseline.

During follow-up, 21 out of 201 patients reached the primary kidney composite outcome and only 5 patients died before reaching the primary outcome. No one reaches ESRD before 57% eGFR decline from the baseline. Univariate analysis showed that the BMI, eGFR, UACR, TNFR1, and PGRN were significantly associated with the risk for renal composite outcome (**Table 4**). The cumulative risk of primary outcome was higher in the group with above-median levels of PGRN than that with below-median levels of it (**Figure 1A**). We also plotted the cumulative incidence rate of the primary outcome per quartile of TNFR1 (**Figure 1B**). The probability of a decline in the GFR was significantly higher in patients with the highest quartile of TNFR1 at baseline than in those with the other lower quartiles of TNFR1. During the median follow-up period of 7.6 years, 17 patients with the above-median of PGRN and 17 patients with the highest quartile of TNFR1 reached the primary outcome (27.6 per 1000 patient-years and 56.9 per 1000 patient-years, respectively, **Table 5**). Univariate Cox regression analysis revealed that the prognostic impact of the serum PGRN or TNFR1 levels on the secondary outcome were not associated with anything except age.

**Table 4:** Cox proportional hazard analysis of risk for primary composite renal outcome in a univariate model.

	Hazard ratio	95% CI	*p*
Male	1.17	0.45–3.01	0.75
Age	0.99	0.95–1.03	0.70
BMI	1.12	1.01–1.24	0.03
SBP	1.02	0.99–1.05	0.16
DBP	0.96	0.93–1.01	0.08
HbA1c	1.20	0.78–1.85	0.40
eGFR (log 1SD = 0.37)	0.33	0.32–0.33	<0.0001
UACR (log 1SD = 2.02)	4.43	2.52–7.78	<0.0001
TNFR1 (log 1SD = 0.40)	5.38	5.26–5.50	<0.0001
PGRN (log 1SD = 0.26)	2.50	2.47–2.52	<0.0001

Abbreviations used in this table are the same as in **Table 1**.

**Table 5:** Incidence of primary kidney composite outcome according to quartiles of baseline circulating PGRN and TNFR1 concentrations.

Quartile	Patients (n)	Incidence per 1000 person-years (No. of event)	
		PGRN	TNFR1
Q1	50	2.9 (1)	2.9 (1)
Q2	50	8.0 (3)	2.7 (1)
Q3	50	22.4 (7)	6.2 (2)
Q4	51	33.0 (10)	56.9 (17)
P for trend		0.002	<0.0001

### Association of Clinical Factors and Biomarkers With Outcome: Multivariate Analysis

As shown in **Table 6**, multivariate analysis revealed that both TNFR1 (HR 4.40, 95% CI 1.60–12.12) and PGRN (HR 1.84, 95% CI 1.04–3.25) were independently associated with the renal composite outcome, even after adjusting for relevant factors and excluding the other biomarker. However, when both TNFR1 and PGRN were added to the same model, only TNFR1 (HR 3.42, 95% CI 1.18–9.89), and not PGRN (HR1.48, 95% CI 0.79–2.77), was the predicted renal composite outcome.

**Table 6:** Cox proportional hazard analysis of the risk for primary composite renal outcome according to clinical predictors and biomarkers in multivariate models.

	Hazard Ratio	95% CI	*p*
TNFR1 (log 1SD = 0.40)			
Adjusted for age, sex, BMI, eGFR, and UACR	4.40	1.60–12.12	<0.01
Adjusted for age, sex, BMI, eGFR, UACR, and PGRN	3.42	1.18–9.89	0.02
PGRN (log 1SD = 0.26)			
Adjusted for age, sex, BMI, eGFR, and UACR	1.84	1.04–3.25	0.04
Adjusted for age, sex, BMI, eGFR, UACR, and TNFR1	1.48	0.79–2.77	0.22

Abbreviations used in this table are the same as in **Table 1**.

## Discussion

In the present study, we investigated the role of PGRN as a prognostic biomarker of kidney function decline in Japanese patients with type 2 diabetes. This study demonstrated that high levels of serum PGRN are associated with eGFR decline independently of baseline eGFR and albuminuria. However, these associations were no longer statistically significant after further adjustment for TNFR1, probably due to the small size of the study sample.

Tang et al. (24) demonstrated that PGRN-deficient mice are susceptible to collagen-induced arthritis, an experimental model of rheumatoid arthritis, and that the arthritic process is reversed by the administration of PGRN. PGRN binds to TNFR and inhibits downstream inflammatory signaling pathways in various diseases. PGRN is a glycoprotein of approximately 75–80 kDa that undergoes proteolysis by matrix metalloproteinases and elastase into small homologous subunits, i.e., constituent granulin peptides (GRNs) (25). In the kidney, recombinant PGRN can prevent renal ischemia/reperfusion injury by attenuating hypoxia-induced inflammatory actions (26). Zhou et al. (27) reported that the expressions of PGRN were significantly reduced in the kidneys of diabetic mice and patients with biopsy-proven DKD compared with those of healthy controls. In addition, they demonstrated that PGRN protects against podocyte injury by regulating mitochondrial homeostasis (28) and autophagy (29). Although PGRN seemed to act as a reno-protective agent in the mice models of several kidney diseases, a negative correlation was also observed between the serum levels of PGRN and eGFR in the present study. Therefore, increased serum PGRN levels may reflect its increased production from somewhere in the body or an increase due to its decreased renal excretion as a compensatory change in patients with diabetes and decreased renal function. Moreover, full-length PGRN generally has an anti-inflammatory effect, whereas its constituent GRNs may have the opposite effect (30). PGRN is highly expressed in macrophages (31) and neutrophils (32) and is proteolytically cleaved by neutrophil elastase (32), proteinase 3 (25), MMP-12 (matrix metalloproteinase 12) (33), and ADAMTS-7 (a disintegrin and metalloproteinase with thrombospondin motifs 7) (34). One hypothesis is that elevated PGRN levels may reflect increased pro-inflammatory GRN levels because PGRN and GRN are indistinguishable from each other in ELISA. In this context, increased serum concentrations of PGRN may only reflect tissue inflammation. Although plausible, the association of CRP with PGRN was not maintained in our stepwise multiple regression analysis. The contradictory effects of PGRN may be caused not only by the pro-inflammatory effect of cleaved PGRN, i.e., GRNs, but also by the presence of its different binding receptors. The PGRN-binding cell membrane receptors, such as TNFRs (TNFR1, TNFR2), DR3, sortilin, toll-like receptor 9 (TLR9), notch receptors, and EphA2 (35), have different characteristics and have been linked to a host of physiological processes and diverse pathological states. Although the exact role of TNFRs is not yet understood, soluble TNFRs may function as decoys for TNFα (36). In the present study, serum PGRN concentrations were correlated mildly with TNFR1 concentrations; however, it should be noted that other research groups have not found any direct physical or functional interaction between PGRN and TNFRs (37, 38).

In the present study, multiple regression analysis revealed that the serum PGRN level was positively associated with BMI, which indicated a relationship between the serum PGRN level and obesity. PGRN also has proinflammatory effects in obesity and insulin-resistant diabetes. Matsubara et al. (39) reported that mice fed with a high-fat-diet (HFD) had increased PGRN levels in white adipose tissue. PGRN exerted pro-inflammatory functions in white adipose tissue of mice with HFD-induced insulin resistance and obesity. Increased levels of PGRN in subcutaneous adipose tissue and serum were associated with obesity, type 2 diabetes, and dyslipidemia (40). In particular, high levels of PGRN inhibit insulin signaling and glucose uptake both *in vitro* and *in vivo* and may be involved in the development of obesity-associated insulin resistance, while deficiency of PGRN provides protective action in this condition (41). In addition, our previous study demonstrated that PGRN deficiency exacerbated renal inflammation despite improving systemic inflammation in adipose tissue in mice with HFD-induced obesity (42). Therefore, PGRN may have two absonant effects, depending on the disease stage and tissue microenvironment. Although obesity generally increases the risk of neurodegenerative diseases, PGRN deficiency is associated with body weight reduction in HFD mice and frontotemporal dementia in both mice and humans (8). The neurometabolic effect of PGRN seems to be ambiguous.

PGRN may aid in the diagnosis, disease activity, and prognosis of various diseases. Yamamoto et al. (43) showed that high-serum PGRN concentrations are associated with poor prognosis in patients with diffuse large B cell lymphoma. Tanaka et al. (44) found that serum PGRN levels were significantly correlated with disease activity in patients with systemic lupus erythematosus, and decreased after successful treatment. However, our literature search failed to find reports evaluating the value of PGRN as a renal prognostic marker. Both advanced CKD and high levels of inflammatory markers have been reported to be associated with poor life prognosis. Indeed, we reported that elevated TNFR levels in sera were associated with the risk of cardiovascular and all-cause mortality in patients undergoing hemodialysis (45). Therefore, we investigated the association between all-cause death and PGRN or TNFR1 in the present study. However, age was only associated with all-cause mortality. The low event rate in pre-dialysis patients compared to patients undergoing dialysis may have influenced these negative associations.

Our findings should be considered in light of the limitations of our study, which are as follows. Firstly, this study was conducted at a single institution with a limited racial demographic. Therefore, it is unknown whether these results can be generalized to other ethnicities, although a number of positive associations between circulating TNFR levels and GFR decline in Caucasian patients with diabetes have been reported so far (46–48). Therefore, the present study confirmed and extended previous studies, which indicated that TNFR1 is a promising biomarker for eGFR decline, even in Japanese patients with type 2 diabetes. Secondly, we measured the biomarkers only once (at the time of enrolment); thus, temporal changes in PGRN levels, with progression to renal insufficiency or any treatments were not considered in the present study. Lastly, although the initial difference between baseline PGRN levels and renal function decline was statistically significant, it failed to remain so after further adjustment for TNFR1 because the size of our study sample was relatively small. A larger study sample will be required to reliably assess the ability of PGRN as a predictor of renal function decline.

In conclusion, PGRN, which is a ligand of TNFR, was also associated with renal function decline independently of the baseline GFR and albuminuria, although its predictive ability was not as strong as that of TNFR1. These results suggest that TNF-related inflammation may be partly associated with the progression of DKD.

## Data Availability Statement

The raw data supporting the conclusions of this article will be made available by the authors, without undue reservation.

## Ethics Statement

The studies involving human participants were reviewed and approved by Institutional Review Board of Juntendo University. The patients/participants provided their written informed consent to participate in this study.

## Author Contributions

MM and TG contributed to conception and design of the study. MM, HS, TS, CK, SI, EA, and TK organized the database. MM and TG performed the statistical analysis. MM wrote the first draft of the manuscript. TG wrote sections of the manuscript. NK and YS reviewed and editing. All authors contributed to manuscript revision, read, and approved the submitted version.

## Funding

This research was partially funded by Grant-in-Aid for Scientific Research (C) grant number 20K08617 to TG.

## Conflict of Interest

The authors declare that the research was conducted in the absence of any commercial or financial relationships that could be construed as a potential conflict of interest.

## Publisher’s Note

All claims expressed in this article are solely those of the authors and do not necessarily represent those of their affiliated organizations, or those of the publisher, the editors and the reviewers. Any product that may be evaluated in this article, or claim that may be made by its manufacturer, is not guaranteed or endorsed by the publisher.
